# Neuroinflammation and Lysosomal Abnormalities Characterise the Essential Role for Oxidation Resistance 1 in the Developing and Adult Cerebellum

**DOI:** 10.3390/antiox13060685

**Published:** 2024-06-03

**Authors:** Eboni M. V. Bucknor, Errin Johnson, Stephanie Efthymiou, Javeria R. Alvi, Tipu Sultan, Henry Houlden, Reza Maroofian, Ehsan G. Karimiani, Mattéa J. Finelli, Peter L. Oliver

**Affiliations:** 1 Mammalian Genetics Unit, MRC Harwell Institute, Harwell Campus, Oxfordshire OX11 0RD, UK; 2 The Dunn School of Pathology, University of Oxford, Oxford OX1 3RE, UK; 3 Department of Neuromuscular Diseases, UCL Queen Square Institute of Neurology, London WC1B 5EE, UK; 4 Department of Pediatric Neurology, Children Hospital, University of Child Health Sciences, Lahore 54660, Pakistan; 5 Molecular and Clinical Sciences Institute, St. George’s University of London, Cranmer Terrace, London SW18 0RE, UK; 6 Department of Medical Genetics, Next Generation Genetic Polyclinic, Mashhad 009851, Iran; 7 School of Medicine, Biodiscovery Institute, University of Nottingham, Nottingham NG7 2RD, UK

**Keywords:** oxidative stress, lysosome, cerebellum, neuroinflammation, ataxia

## Abstract

Loss-of-function mutations in the TLDc family of proteins cause a range of severe childhood-onset neurological disorders with common clinical features that include cerebellar neurodegeneration, ataxia and epilepsy. Of these proteins, oxidation resistance 1 (OXR1) has been implicated in multiple cellular pathways related to antioxidant function, transcriptional regulation and cellular survival; yet how this relates to the specific neuropathological features in disease remains unclear. Here, we investigate a range of loss-of-function mouse model systems and reveal that constitutive deletion of *Oxr1* leads to a rapid and striking neuroinflammatory response prior to neurodegeneration that is associated with lysosomal pathology. We go on to show that neuroinflammation and cell death in *Oxr1* knockouts can be completely rescued by the neuronal expression of Oxr1, suggesting that the phenotype is driven by the cell-intrinsic defects of neuronal cells lacking the gene. Next, we generate a ubiquitous, adult inducible knockout of *Oxr1* that surprisingly displays rapid-onset ataxia and cerebellar neurodegeneration, establishing for the first time that the distinctive pathology associated with the loss of *Oxr1* occurs irrespective of developmental stage. Finally, we describe two new homozygous human pathogenic variants in *OXR1* that cause neurodevelopmental delay, including a novel stop-gain mutation. We also compare functionally two missense human pathogenic mutations in *OXR1,* including one newly described here, that cause different clinical phenotypes but demonstrate partially retained neuroprotective activity against oxidative stress. Together, these data highlight the essential role of *Oxr1* in modulating neuroinflammatory and lysosomal pathways in the mammalian brain and support the hypothesis that OXR1 protein dosage may be critical for pathological outcomes in disease.

## 1. Introduction

A family of proteins that have been gaining increasing attention in recent years share the highly conserved Tre2/Bub2/Cdc16 (TBC), lysin motif (LysM) and domain catalytic (TLDc) domain [1]. The domain is comprised of approximately 200 amino acids, with highly conserved residues present in all eukaryotic TLDc proteins, including those from plants and yeast [2]. In humans, the TLDc protein family is composed of six members: oxidation resistance 1 (OXR1 or TLDC3), nuclear receptor coactivator 7 (NCOA7 or TLDC4), TBC/LysM-associated domain containing 1 (TLDC1 or MEAK7), TBC1 domain family member 24 (TBC1D24 or TLDC6), TBC/LysM-associated domain containing 1 (TLDC1 or MEAK7) and TBC/LysM-associated domain containing 2 (C20ORF118 or TLDC2), with the more evolutionary distant interferon-induced protein 44 (IFI44 or TLDC5). The three-dimensional structure of the TLDc domain has been solved, revealing four alpha-helices and ten beta-strands [3,4], yet it contains no structural similarity to any known protein folds, providing limited functional clues.

Interest in TLDc proteins has, in part, been driven by the discovery of multiple pathogenic mutations in a range of human neurodevelopmental disorders with some overlapping clinical features. For example, predominantly predicted loss-of-function variants in *TBC1D24* cause a group of disorders characterised by childhood-onset epilepsy and hearing loss, with neurodegeneration in some cases [5]. In addition, a homozygous null allele for *NCOA7* was discovered to be associated with autism [6]. More recently, three independent biallelic variants were discovered: *OXR1*, the TLDc gene most closely related to *NCOA7* (also summarised in figure below) [7]. All these individuals were described with hypotonia, developmental and speech delay, intellectual disability, epilepsy and cerebellar atrophy; a protein analysis of fibroblasts cultured from two individuals suggests a total loss of OXR1 protein expression [7]. Similarly, a fourth familial *OXR1* homozygous variant has since been discovered with highly similar clinical features, with evidence that this is also a complete loss-of-function variant due to defective splicing in the TLDc domain [8]. All three of these TLDc proteins are highly expressed in the nervous system and have been implicated in multiple neurodevelopmental and cellular survival pathways [8,9,10,11,12,13]. Yet it is still unclear how their molecular function relates to disease and why loss-of-function variants appear to result in similar clinical outcomes.

Much of the functional studies of TLDc proteins have focussed on a role in the oxidative stress response. The stems from the first description of OXR1 as a protein protective against oxidative DNA damage in *E. coli* [14]. Subsequently, this apparent oxidative stress-related function was shown to be evolutionary conserved, with the finding that the yeast strain *S. cerevisiae* with the deletion of the homologue *scOxr1* was more sensitive to hydrogen peroxide (H_2_O_2_) treatment than controls [14]; interestingly, this phenotype could be rescued by expressing human OXR1 targeted to the mitochondria [15]. Further functional work has demonstrated a similar role in mammalian systems (reviewed in [16]). Indeed, OXR1 over-expression has proved to be effective against oxidative stress-associated cellular damage and death in a range of disease model systems, from motor neuron disease and retinal degeneration to spinal cord injury [17,18,19,20]. Loss-of-function studies in vitro have indicated molecular pathways such as p53 signalling might underlie this conserved activity [21], alongside the direct or indirect modulation of genes that increase cellular resistance to oxidative stress, including antioxidant enzymes [22]. OXR1 is expressed as multiple isoforms, with mammals typically expressing one or more ‘full-length’ proteins containing the TLDc domain, along with a shorter C-terminal isoform that is made up almost exclusively of the TLDc region (also see figure below) [16]. In mice, a homozygous spontaneous deletion mutant was discovered where the entire coding sequence of *Oxr1* and the adjacent gene actin-binding Rho-activating protein (*Abra*) was removed. These animals display degeneration in the cerebellar granule cell layer from postnatal day (P)19, followed by rapidly progressive ataxia and death by P24 [23]. This phenotype was fully rescued by ubiquitous expression of an *Oxr1* transgene, confirming that the phenotype is caused by the loss of the gene [23]. A second insertional mouse mutant was then created, in which the last 101 amino acids of the TLDc domain of Oxr1 was removed; this mutant has an essentially identical phenotype to the Oxr1 deletion mouse, demonstrating the importance of the TLDc domain for the function of the protein [2]. The fact that both the full-length and shorter C-terminal isoform is disrupted in this *Oxr1* knockout model is significant, as multiple studies have demonstrated that the TLDc region alone confers the evolutionary-conserved oxidative stress resistance functionality [15]. In addition, loss-of-function mutants in *Drosophila* can be phenotypically rescued with even the shortest mammalian TLDc domain-containing isoforms [7]. Moreover, recent work in the mouse has deleted exclusively a full-length *Oxr1* brain-expressed isoform, resulting in a much less severe phenotypic outcome than the complete null allele [24]. Somewhat unexpectedly, these particular mutants are characterised by increased growth hormone secretion and a fatty liver, with Oxr1 regulation of an arginine methyl-transferase implicated as a key molecular mechanism [24].

Together, these studies demonstrate the complex and multifaceted roles of OXR1; yet how disrupting the gene causes the very specific pathogenic features in disease is not known, nor is how this relates to suggested antioxidant functions. Here, we have investigated this question by developing and analysing several independent mice and human genetic model systems, highlighting the functional significance of OXR1 in inflammatory and lysosomal pathways that underlie the distinctive neuropathological features shared with loss-of-function clinical outcomes.

## 2. Materials and Methods

### 2.1. Mouse Strains

All breeding and animal procedures were performed under project licence PP3246997 with local ethical approval, compliant with the Animals Scientific Procedures Act 1986 and in accordance with UK Home Office regulations. All colonies were bred and maintained on a C57BL/6J background. Mice were housed in individually ventilated cages (IVCs) with ad libitum access to food and water. IVCs were limited to a maximum of 5 mice per cage and kept on a 12 h on:off light cycle. Conditional ready *Oxr1^tm1c^* allele and *Oxr1^tm1d^* knockout allele were derived from the original Oxr1 knockout-first (tm1a) gene trap cassette (as described in [25]) inserted into the TLDc domain, which targets deletion of all isoforms of Oxr1 [2]. For generation of inducible KO animals, *Oxr1^tm1c^* mice were crossed to the tamoxifen-inducible *B6.Cg-Tg(UBC-cre/ERT2)1Ejb/J Cre* line [26] to generate experimental cohorts. Tamoxifen (Merck, Gillingham, UK) was dissolved to a concentration of 20 mg/mL in filter sterilised oil (Merck, Gillingham, UK) (2% ethanol) at 42 °C and then stored at 4 °C in the dark. *Oxr1^tm1c/tm1c^/UBC-Cre* experimental cohort mice were dosed at 200 mg/kg for five consecutive days by oral gavage and left to recover for at least 14 days. For genetic rescue experiments, heterozygous *Oxr1^tm1d^* mice were crossed with mice that were hemizygous for the Prnp-Oxr1 transgene (Tg) to produce experimental cohorts on a C57BL/6 J background.

### 2.2. Phenotyping

Female mice (n = 9–12 per genotype) were subjected to an accelerating rotarod performance test at three time points. The device (Ugo Basile, Gemonio, Italy) was set to accelerate from 4 to 40 rpm over 300 s. Mice were placed on the rod, opposing the direction of rotation. A rotarod session consisted of a single trial each day on three consecutive days. The trial ended, and the time was taken when a mouse exceeded two passive rotations or fell from the rod (latency to fall) for the three trials at each time point; the average time of three trials was calculated.

### 2.3. Tissue Immunohistochemistry and TUNEL Staining

Mice were perfused with 4% paraformaldehyde, and brains were processed into wax blocks for cutting at 10 µM. IBA1 immunostaining was carried out using the Vectorstain Elite ABC DAB kit (Vectorlabs, Newark, CA, USA) using anti-IBA1 019-19741 (Wako, Neus, Germany) at 1:1000 dilution overnight at 4 °C. Terminal deoxynucleotidyl transferase dUTP nick end labelling (TUNEL) was carried out on 12 µm un-fixed, frozen brain sections in OCT (Merck, Gillingham, UK) mounted on Superfrost Plus slides (Thermo Fisher Scientific, Waltham, MA, USA). Experiments were carried out using the in situ cell death detection kit, Fluorescein (Merck, Gillingham, UK), following the manufacturer’s protocol. Slides were mounted using ProLong Gold Antifade Mountant with DAPI (Thermo Fisher Scientific, Waltham, MA, USA). Imaging was carried out using a Zeiss LSM 700 inverted confocal microscope (Carl Zeiss, Cambridge, UK) and analysed using Zen Black 2.1. At least three animals of both sexes per genotype were examined for these studies.

### 2.4. Immunocytochemistry

Cells were fixed using 4% paraformaldehyde (PFA) in PBS for 20 min at room temperature (R/T) and then washed three times in PBS. Cells were blocked in 10% normal goat serum (NGS) in PBS with 0.5% Triton X-100 (PBS-Tx) and incubated with primary antibodies in 3% NGS in PBS-Tx overnight at 4 °C. Coverslips were washed with PBS before AlexaFluor secondary antibody (Thermo Fisher Scientific, Waltham, MA, USA) incubation for 2 h at R/T in the dark. Coverslips were washed and mounted in ProLong Diamond Antifade Mountant with DAPI (Thermo Fisher Scientific, Waltham, MA, USA) and imaged using a Zeiss inverted confocal microscope as above.

### 2.5. RNA Extraction, cDNA Synthesis and qPCR

Total RNA was extracted from cerebellar tissue using the Maxwell RSC Instrument and simplyRNA Tissue Kit (Promega, Madison, WI, USA) as per manufacturer’s instructions. RNA quantification and integrity were established using a Bioanalyzer (Agilent, Santa Clara, CA, USA). cDNA was synthesised using 2 µg RNA per reaction and the High-Capacity cDNA Reverse Transcription Kit (Thermo Fisher Scientific, Waltham, MA, USA). Moreover, 20 µL reactions were set up in triplicates with 20 ng cDNA, 5 µM primers and 10 µL Fast SYBR Green Master Mix (Thermo Fisher Scientific, Waltham, MA, USA) per well in a 7500 Fast Real-Time System PCR machine (Thermo Fisher Scientific, Waltham, MA, USA). The amplification reaction protocol was established as 1 cycle at 95 °C for 20 s, 40 cycles at 95 °C for 3 s and 60 °C for 30 s. A melt dissociation curve was established to determine that only one PCR product was amplified (heating ramp from 55 to 95 °C). All reactions were normalised to internal control housekeeping genes glyceraldehyde 3-phosphate dehydrogenase (*Gapdh*), 40 S ribosomal 76 protein S16 (S16) and peptidylprolyl isomerase A (*Ppia*) and fold changes were calculated using ΔΔ Ct method and expressed relative to a control genotype. Primers used for qPCR, shown as forward Sequence (5′–3′)/reverse sequence (5′–3′):

*C1qa* AAAGGCAATCCAGGCAATATCA/TGGTTCTGGTATGGACTCTCC
*Ctsd* GCTTCCGGTCTTTGACAACCT/CACCAAGCATTAGTTCTCCTCC
*Cd68* TGCCTGACAAGGGACACTTC/TGGTGGCTTACACAGTGGAC
*Gapdh* CGGCCGCATCTTCTTGTG/CCGACCTTCACCATTTTGTCTAC
*Gfap* GCAGAAGCTCCAAGATGAAACC/CGAACTTCCTCCTCATAGATCTTC
*Hexb* AATGGTCAGCCGTGGAATAG/CATAGCTGGAATGCTGTAGACG
*Laptm5* GATGCCGTACCTCAGGATGG/CTCCCGGTTCTTGACCACG
*Ly86* TATACTATGCCGGCCCTGTC/GGGTCCCCTGAGATTGAGTT
*Ppia* AGTTTTTTATCTGCACTGCCAAGA/CCTTCCCAAAGACCACATGCT
*S16* TTCTGGGCAAGAGCGATT/GATGGACTGTCGGATGGCA
*Tgfβ* TGAGTGGCTGTCTTTTGACG/GGTTCATGTCATGGATGGTG
*Trem2* CTGGAACCGTCACCATCACTC/CGAAACTCGATGACTCCTCGG

### 2.6. RNA Sequencing

Total RNA was extracted from snap-frozen male *Oxr1* WT and KO whole cerebellar tissue at P19 (n = 7 WT, n = 5 KO, littermate WT/KO groups from five independent litters) using the Maxwell RSC Instrument (Promega, Madison, WI, USA) with DNase treatment. Bioanalyzer RNA 6000 Nano assay (Agilent) was used to assess RNA integrity and quantity and run on the Bioanalyzer 2100 system (Agilent Technologies, Santa Clara, CA, USA). Samples were sent to Cambridge Genomic Services (University of Cambridge, UK) for sequencing library preparation and next-generation sequencing. Downstream analysis to detect differential gene expression was performed by Cambridge Genomic Services. TruSeq Stranded Total RNA library preparation kit (Illumina, San Diego, CA, USA) was used to generate sequencing libraries and index coding, as per manufacturer’s instructions. Cluster generation of index-coded samples was performed on PE Cluster Kit cBot-HS (Illumina) using the cBot Cluster Generation System (Illumina, San Diego, CA, USA), and single-end sequencing was carried out with the NextSeq500 platform (Illumina, San Diego, CA, USA). A total of 31 to 40 million reads per sample was achieved. Quality control of raw data was performed using FastQC software (v0.11.4), and reads were trimmed using TrimGalore (v0.5.0). Spliced Transcripts Alignment to a Reference (STAR) (v2.7.1) was utilised to map reads to Ensembl Mus_musculus GRCm38 (release 99) reference genome. HTSeq (v.0.6.1) software was applied to calculate the number of reads that map to reference genome. Pairwise differential gene expression analysis was performed between genotypes using counted reads and edgeR (v3.26.5, R v.3.6.1); the Benjamini–Hochberg method was used to adjust *p*-values for multiple testing of the FDR. Subsequent Gene Ontology (GO) enrichment analysis of DEGs (FDR ≤ 0.01) was performed using ordered queries in g: Profiler and GOrilla (Gene Ontology enRIchment anaLysis and visuaLizAtion tool). A complete set of processed, normalised data are provided in Supplementary Table S1. Raw, unprocessed data files are available as NCBI BioSample accession numbers SAMN40425656 to SAMN40425667.

### 2.7. Protein Extraction, SDS-PAGE and Western Blotting

Tissue samples were cut into small pieces and placed into BioPulverizer Lysing Matrix D tubes (MP Biomedicals, Irvine, CA, USA) with cold radioimmunoprecipitation assay buffer with cOmplete Mini EDTA-free protease and phosphatase inhibitors (Roche) (RIPA + PPI). Samples were homogenised for 2 × 15 s in a FastPrep-24 Instrument (MP Biomedicals, Irvine, CA, USA), spun down for 10 min, 8000× *g* at 4 °C. Cells were scraped and collected in cold RIPA + PPI buffer. Lysates were incubated on a rotator at 4 °C for 30 min and spun down for 10 min, 8000× *g* at 4 °C. SDS-PAGE and Western blotting protein samples were thawed on ice and quantified using the DC Protein Assay (Bio-Rad, Hercules, CA, USA) method and bovine serum albumin (BSA) protein standards. Samples were mixed with 5 × NuPAGE loading dye (Thermo Fisher) and 10 × NuPAGE reducing agent (Thermo Fisher), then heat denatured. Samples were run on NuPAGE Bis-Tris gels (Thermo Fisher) in MOPS or MES buffer (Thermo Fisher), followed by dry transfer using an iBlot Gel Transfer system and nitrocellulose iBlot Transfer Stacks (Thermo Fisher Scientific, Waltham, MA, USA). Membranes were blocked in 4% milk in PBS + 0.1% Tween (PBS-T). Primary antibodies were incubated rocking overnight at 4 °C. Membranes were then washed in PBS-T and incubated rocking with IRDye secondary antibodies (LI-COR, Lincoln, NE, USA) for 1 h at R/T. Membranes were washed twice in PBS-T and once in PBS before drying. Membranes were imaged using LI-COR Odyssey CLx, and 80 were analysed using Image Studio Lite Version 5.2 Software (LiCor Biosciences, Lincoln, NE, USA). Western blots were probed with anti-OXR1 (Proteintech 13514-1-AP 1:1000) or anti-alpha-tubulin (Merck, Gillingham, UK T9026 1:3000).

### 2.8. Electron Microscopy

For TEM preparations, *Oxr1* WT and KO littermates (n = 3; 2 male, 1 female each genotype) were perfused at P19 with saline and then 2.5% glutaraldehyde + 2% PFA in 0.1 M sodium cacodylate buffer. Cerebella were cut into 1 mm cubed blocks from the midline using a brain blade matrix. Samples were then loaded into a Leica EM AMW automated microwave processing unit and processed into resin. The controlled application of microwaves during processing improves reagent penetration and considerably shortens incubation times [27]. In the AMW, samples were washed 4 times with 0.1 M sodium cacodylate buffer, including one round with 50 mM glycine to block free aldehydes. The samples were then stained with 1% osmium tetroxide with 1.5% potassium ferrocynaide, washed with water, stained with 2% uranyl acetate, then taken through an ethanol dehydration series and infiltrated with low-viscosity epoxy resin (TAAB Laboratories). Samples were removed from the AMW and then given an additional overnight incubation in fresh resin with rotation at room temperature. Samples were then embedded in beem capsules and polymerised for 48 hr at 60 °C. Ultrathin sections (90 nm) were obtained using a Diatome diamond knife on a Leica EM UC ultramicrotome and transferred to 200 mesh copper grids. Sections were stained for 5 min with Reynold’s lead citrate. Images were acquired using a FEI Tecnai T12 TEM operated at 120 kV and a OneView CMOS camera (Gatan, Pleasanton, CA, USA).

### 2.9. Human OXR1 Variant Constructs and Assays

The DNA construct pcDNA3-OXR1-FL-HA WT was derived from the human sequence NM_001198533 with a C-terminal in-frame HA-tag cloned into pcDNA3 using KpnI and XbaI sites (NEB). Mutant clones were generated by site-directed mutagenesis (Quikchange, Agilent Technologies, Santa Clara, CA, USA). Mutagenesis primers used were as follow: hOXR1 S345P F: CAGAATCAGAACTTCCCCCTATACGAGAG; hOXR1 S345P R: CTCTCGTATAGGGGGAAGTTCTGATTCTG; hOXR1 F K78R: GACACTGGCCAAAGGAAGACCCTAGAC; hOXR1 R K78R: GTCTAGGGTCTTCCTTTGGCCAGTGTC. Mutations were confirmed by Sanger sequencing (Source Bioscience, Cambridge, UK). After 48 h of transfection, cells cultured in 10 cm dishes were washed twice with PBS and lysed with RIPA buffer (150 mM NaCl, 1% Triton-X, 0.5% sodium deoxycholate, 0.1% SDS, 50 mM Tris-HCl pH 7.5) complemented with a cocktail of protease inhibitors (Merck, Gillingham, UK). After 30 min incubation on ice, lysates were cleared by centrifugation (30 min, 4 °C, 16,000× *g*). Protein concentration was determined by BCA assay (ThermoFisher). Laemmli buffer (ThermoFisher) containing beta-mercaptoethanol (ThermoFisher) was added to 65 μg protein extracts and boiled for 5 min. Protein extracts were run on 4–12% pre-cast gels (ThermoFisher). Western blots were probed with anti-HA (Merck H6908-100UL 1:2000) or anti-Gapdh (ProteinTech 60004-1-Ig 1:1000) and with secondary antibody anti-HRP rabbit or mouse (Thermo Fisher Scientific, Waltham, MA, USA) using ELC Prime (Merck, Gillingham, UK) with an ImageQuant LAS4000 (GE Healthcare, Amersham, UK).

### 2.10. Cell Culture and Treatment

Human neuroblastoma cell lines (SH-SY5Y cells) were cultured in DMEM supplement containing Glutamax with 10% FBS. Cells were transiently transfected using Lipofectamine 3000 as per the manufacturer’s instructions (ThermoFisher). One day after transfection, cells were treated with H_2_O_2_ (Sigma) at 600 μM (for ROS production assay) or 1200 μM (for cell death assay) for 24 h.

### 2.11. Reactive Oxygen Species (ROS) Production Assay

Intracellular ROS levels were determined using DCFDA/H2DCFDA cellular ROS assay kit (Abcam, Cambridge, UK) following the manufacturer’s instructions; cells were cultured, transfected and treated in 96-well plates (black, clear bottom). Briefly, cells were washed once with the provided 1× buffer, which was replaced with 1× buffer containing 20 μM DCFDA. Cells were incubated at 37 °C for 45 min, protected from light. After treatment, DCFDA-containing solution was aspirated and replaced with 1× buffer; fluorescent signal was read at Ex/Em 485/535 on a FluostarOmega (BMG Labtech, Aylesbury, UK) plate reader.

### 2.12. Cell Death Assay

Cell death was determined by propidium iodide (PI) staining. Cells were cultured and transfected in 96-well plates (black, clear bottom). After 24 h of treatment, cells were gently washed once with PBS, and 3 µM PI (Molecular probes, diluted in PBS) was added to wells and incubated at 37 °C for 30 min. Fluorescent signal was read at Ex/Em 530/620 on a FluostarOmega (BMG Labtech, Aylesbury, UK) plate reader.

### 2.13. Next Generation Sequencing

Single-nucleotide variations (SNVs) were identified by WES in Patient 1, sequenced on Illumina sequencers as described elsewhere [28]. The bioinformatics filtering strategy included screening for only exonic and donor/acceptor splicing variants. Rare variations present at a frequency above 1% in GnomAD or present from exomes or genomes within datasets from UK Biobank and UK 100,000 genome project or from internal research databases (e.g., Queen Square Genomics and UCL SYNaPS Study Group) were excluded. Candidate variants were then confirmed by Sanger sequencing in all the families. Sequence candidate variants were interpreted according to the ACMG guidelines [29]. Volcano plots were generated using SRplot [30].

### 2.14. Data Analysis and Statistics

FIJI and ZEISS ZEN (Black 2.1 and Blue 3.4 editions) microscopy software were used to process and analyse confocal microscopy images. Western Blot images were analysed using Image Studio Lite Quantification Software 2.0 (LI-COR, Lincoln, NE, USA). All numerical data were analysed using Prism version 9.0 (GraphPad Software, Inc., Boston, MA, USA), and appropriate statistical analysis and post hoc tests were applied (ns ≥ 0.05, * ≤ 0.05, ** ≤0.01, *** ≤ 0.001, **** ≤ 0.0001). Data are represented as mean ± SEM unless otherwise stated in figure legends. Broadly, an unpaired Student’s *t*-test was used for two normally distributed data sets, and one or two-way ANOVA was applied to normally distributed data sets with more than two experimental groups. Alternative analyses are stated in figure legends where used.

## 3. Results

### 3.1. Pre-Symptomatic Lysosomal and Inflammatory Pathology in the Oxr1 KO Cerebellum

**Figure 1:**
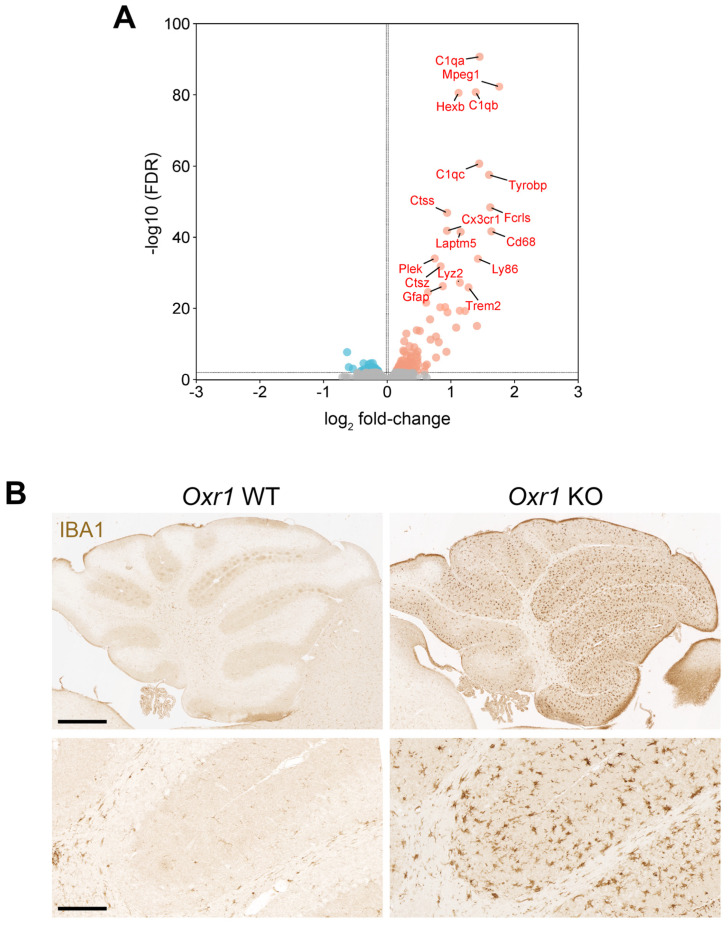
Induction of lysosomal and inflammatory pathways in the *Oxr1* KO cerebellum. (**A**) Volcano plot of all RNA-seq data indicating the most significantly upregulated genes (red) and downregulated (blue) in the *Oxr1* KO versus WT cerebellum tissue plotted as log_2_ fold change (FC) versus FDR. Grey dots indicate genes with a non-significant FC. (**B**) Representative IBA1 immunostaining of parasagittal cerebellar sections from WT and KO mice at P22. Scale bars: 300 μm (upper panels) and 60 μM (lower panels).

First, we wanted to investigate the molecular mechanisms underpinning cerebellar dysfunction and disease progression in the constitutive *Oxr1* knockout (KO) mouse [2,23]. The neuropathological time-course in this mutant is rapid but well-established and robust, with ataxia detectable from postnatal day (P)20–22. At P19, no oxidative DNA damage or cell death is observed in the granule cell layer (GCL) of KO animals; therefore, this time point was chosen to avoid aberrant gene expression from the considerable number of apoptotic cells observed as part of end-stage (P22–24) disease pathology [23]. Whole cerebellar RNA samples from KO and littermate pair wild-type (WT) male mice were subjected to bulk RNA-seq. Analysis of the data revealed 197 significantly differentially expressed (DE) transcripts between the two genotypes (based on a false discovery rate (FDR) < 0.01), with the majority (143 versus 54) of genes over-expressed in the KO cerebellum (complete dataset in Supplementary Table S1). Examining the gene identities using ontology tools and focussing on the transcripts with lowest *p*-values show that two key classes are upregulated, namely genes related to the innate immune response and lysosomal function (Figure 1A, Supplementary Figure S1). For example, genes include members of the complement system (*C1qa-c*) and well-established markers or regulators of glial cell activation such as macrosialin (*Cd68*), macrophage-expressed gene (*Mpeg*), Fc receptor type 2 (*Fcrls*) and Triggering Receptor Expressed on Myeloid Cells 2 (*Trem2*). In addition, genes encoding the enzymes cathepsin D, S, Z (*Ctsd*, *Ctss* and *Ctsz*) and hexosaminidase subunit beta (*Hexb*) that degrade target substrates within lysosomes were also found to be significantly upregulated. There was no clear indication of oxidative stress-related pathways being altered in the KO cerebellum, however. Given the upregulation of neuroinflammatory genes, to then assess the extent of microglial cell activation in the *Oxr1* KO brain, IBA1 immunostaining was carried out at P22. In KO tissue, there was a striking increase in the extent of IBA1-positive immunostaining in the cerebellum, with microglia showing evidence of ramified and activated states in both the GCL and molecular layer (Figure 1B).

**Figure 2:**
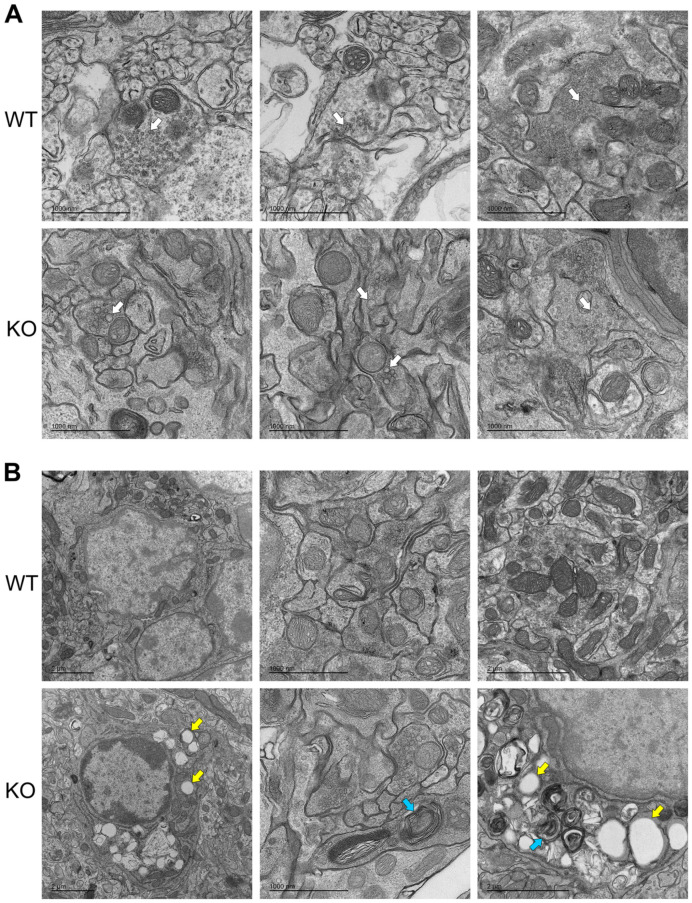
Ultrastructural analysis of P19 cerebellar tissue from WT and *Oxr1* KO mice. (**A**) Representative transmission electron micrographs with white arrows indicating enlarged vesicle structures in KO tissue not present in WT. (**B**) Representative transmission electron micrographs with arrows indicating lipid droplet accumulation (yellow) and myelin figures (blue) in KO cerebellar tissue. Scale bars as indicated on each panel.

Given the transcriptomic data, we were interested in determining whether examining organelle morphological abnormalities at the ultrastructural level might provide more insight into the cerebellar pathology; therefore, transmission electron microscopy (TEM) was carried out from fixed WT and KO cerebellum tissue from littermates taken at P19. Multiple aberrant membrane-associated cellular structures were visible in micrographs from KO mice, such that a pathologist blinded to genotype was able to identify the mutant from WT samples consistently. Examples of distinctive features included enlarged, misshapen vesicle-like structures at synapses (Figure 2A), the accumulation of aberrant membranous cellular whorls known as myelin figures (Figure 2B), in addition to the frequently increased presence of lipid droplets (Figure 2B). It is unclear whether these features are a cause or a consequence of neurodegeneration in the KO cerebellum; however, these data support the hypothesis that the deletion of *Oxr1* influences lysosomal function and intracellular trafficking pathways. Furthermore, the presence of myelin figures is suggestive of disrupted autophagy [31].

### 3.2. Neuronal Expression of Oxr1 Rescues Oxr1 KO Pathology In Vivo

Multiple single-cell transcriptomic studies demonstrate that *OXR1/Oxr1* is expressed in glial cells in the brain as well as many classes of neurons [32], yet it is not known what is driving the neuroinflammatory response and lysosomal signals in the *Oxr1* KO cerebellum we observe. Interestingly, in a *Drosophila* genetic rescue experiment, neuropathology caused by the whole-body deletion of *Mtd* (*Oxr1* orthologue) could be completely rescued by a full-length *Oxr1* cDNA driven by a neuronal promoter [7]. These data suggest that signalling from neurons may be sufficient to instigate pathological effects extrinsic to these cells. Therefore, we carried out a similar experiment in our mammalian system by crossing *Oxr1* KO mice with a transgenic line we have published previously that expresses an HA-tagged mouse full-length *Oxr1* cDNA in neurons specifically [33].

**Figure 3:**
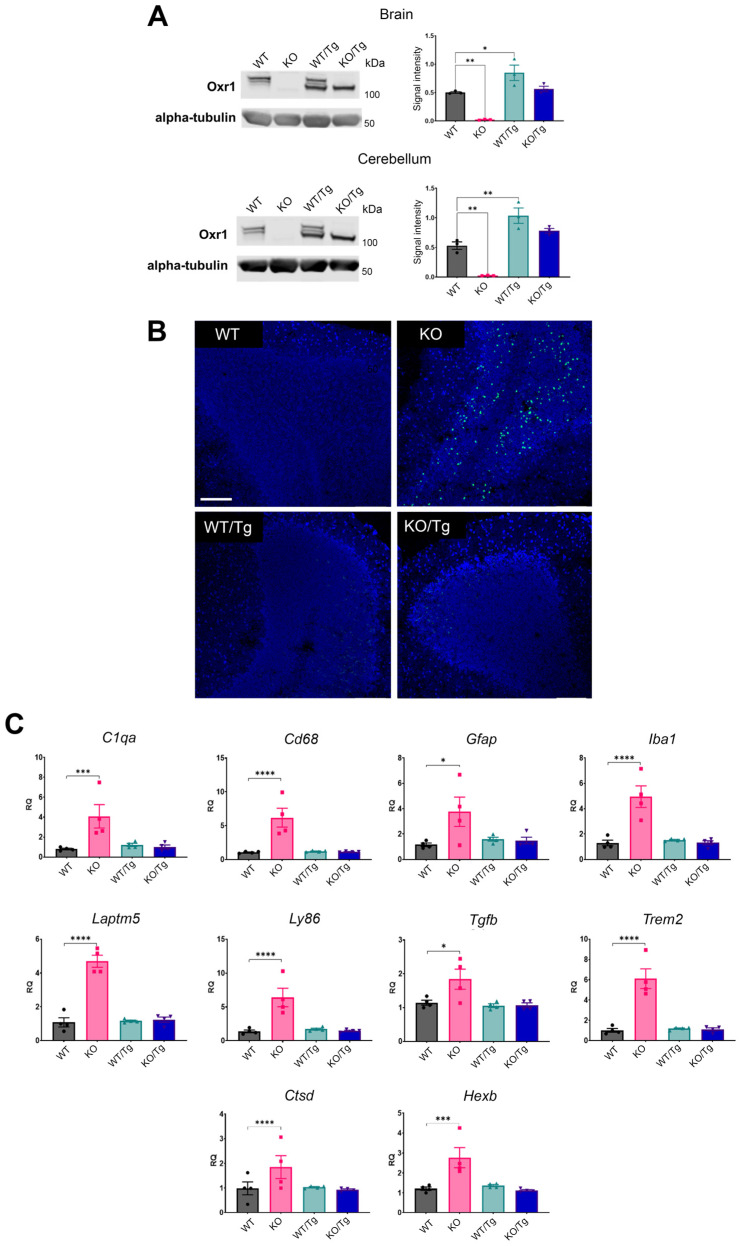
Rescue of neuropathology in the *Oxr1* KO cerebellum by neuronal expression of an OXR1 transgene. (**A**) Quantified Western blotting of total Oxr1 protein levels in the brain and cerebellum from the indicated genotypes; *Oxr1* KO or WT mice with (Tg) or without (WT) expression of the transgene. Signal intensity is normalised to alpha-tubulin. (**B**) Representative TUNEL staining of cerebellar tissue indicating apoptotic cells from the four genotypes indicated. Scale bar: 40 μM. (**C**) qRT-PCR of selected genes from the cerebellum of the indicated genotypes at P22. Data are shown as relative quantification (RQ) to the housekeeping internal control gene. Data are presented as ±SEM relative to WT, 2-way ANOVA, *p*-values: * *p* ≤ 0.05, ** *p* < 0.01, *** *p* < 0.001 **** *p* ≤ 0.0001.

Breeding *Oxr1* KO mice and hemizygous *Oxr1* transgenic mice (Tg) generated experimental animals of four genotypes, namely *Oxr1* KO or WT mice with (Tg) or without (WT) expression of the transgene (KO/WT, KO/Tg, WT/WT and WT/Tg). We confirmed that Oxr1 expression was at approximately physiological levels as compared to the endogenous Oxr1 isoforms found in the mouse brain (Figure 3A). During breeding and welfare assessments, all KO/WT (*Oxr1* KO) animals reached the disease end-point as expected (P22); however, none of the mice of other genotypes—including KO/Tg mice—displayed signs of early-onset ataxia or other adverse in-cage behaviour into adulthood. We, thus, determined whether the transgene was rescuing cerebellar neuropathology in KO mice at P22. Firstly, TUNEL staining on cerebellar sections demonstrated that no cell death was detected in KO/Tg mice at this timepoint compared to the expected pathological profile in KO/WT animals (Figure 3B). Secondly, we selected key differentially expressed genes from the *Oxr1* KO RNA-seq study (Supplementary Table S1) and carried out qRT-PCR to determine whether these molecular pathways were also rescued in the KO/Tg cerebellum at the disease end-point. All of the genes tested that showed dysregulation in *Oxr1* KO mice, including glial cell and complement activation (e.g., *Cd68*, *C1qa*) or lysosomal enzymes (*Hexb*), presented levels in KO/Tg mice similar to levels in WT/WT and WT/Tg controls (Figure 3C). Together, these findings demonstrate that the pathology that characterises constitutive *Oxr1* deletion can be completely rescued by neuronal expression of Oxr1, suggesting that neurons play a key role in instigating neurodegeneration and in the KO cerebellum.

### 3.3. Adult-Onset Deletion of Oxr1 Causes Pathology Equivalent to Constitutive Loss of the Gene

**Figure 4:**
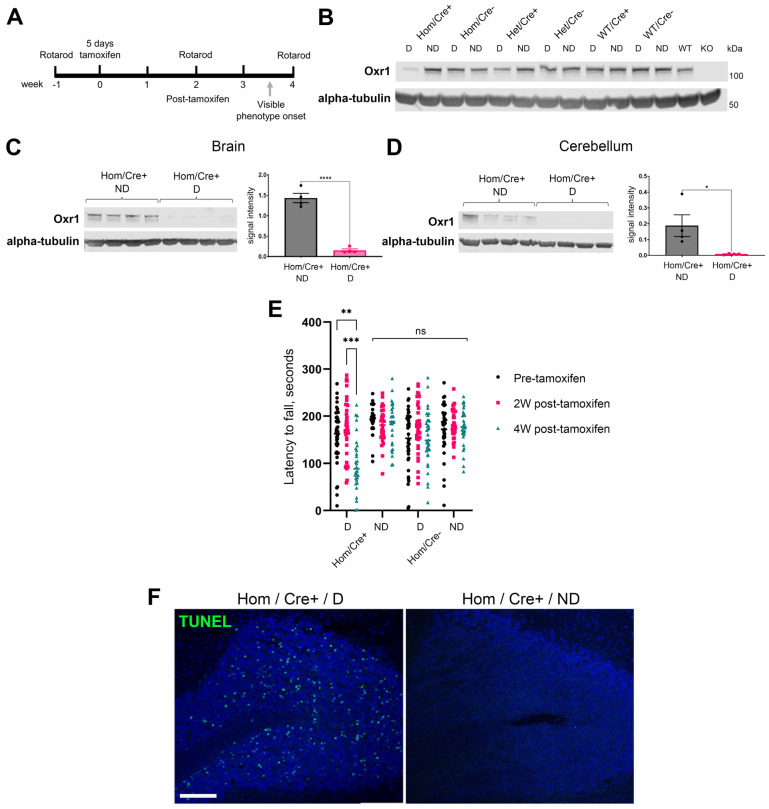
Inducible knockout of *Oxr1* in adult mice results in cerebellar neurodegeneration. (**A**) Timeline of mouse treatment and behavioural assessment. (**B**) Representative Western blotting of Oxr1 from the brain in the indicated genotypes 4 weeks after tamoxifen dosing (D) or no dosing (ND); animals are carrying either two (Hom), one (Het) or no (WT) copies of the floxed Oxr1 allele, with either one (Cre+) or no (Cre-) copies of the CreERT2 transgene. (**C**,**D**) Quantified protein expression of OXR1 in the brain (**C**) and cerebellum (**D**) 4 weeks after tamoxifen treatment, normalised to alpha-tubulin. (**E**) Accelerating rotarod performance as latency to fall over three trials, prior to 2 weeks and 4 weeks after tamoxifen dosing (n = 9–12 per genotype). (**F**) Representative images of TUNEL-positive apoptotic cells localised to the granule cell layer of the cerebellum in a Hom/Cre+ tamoxifen-dosed compared to a representative control Hom/Cre+ non-dosed animal 4 weeks post-treatment. Scale bar: 40 μM. Data are shown as ±SEM. Western blot: 2-way ANOVA, rotarod: mixed-effects analysis, followed by Turkey’s multiple comparisons test. *p*-values: * *p* ≤ 0.05, ** *p* < 0.01, *** *p* < 0.001 **** *p* ≤ 0.0001.

Pathology of the constitutive *Oxr1* KO mouse shares features of human loss-of-function mutations with the early-life onset of cerebellar neurodegeneration [2,7,8]. Importantly, the expression of the gene remains constantly low in utero in both humans and mice before a striking increase in the brain—including the cerebellum—over the early post-natal period [2,34]. As such, we were interested in determining whether there are essential functions of Oxr1 in adult animals that are independent of any specific neurodevelopmental roles. Using a previously described conditional allele of *Oxr1* that disrupts all isoforms of the gene [2], we generated an inducible knockout model in the mouse. Animals homozygous for this conditional construct combined with a single copy of a tamoxifen-inducible, ubiquitous cre-expressing transgene were treated for five days with tamoxifen (Hom/Cre+/dose) alongside a range of control lines for tamoxifen treatment and the presence of cre-recombinase or the conditional allele. Mice were then used for pathological and behavioural analysis (Figure 4A). Hom/Cre+/dose animals show a robust knockdown of the Oxr1 protein 2 weeks after tamoxifen administration, resulting in an almost complete loss in expression in the cerebellum by 4 weeks (Figure 4B–D). Control mice that were dosed with tamoxifen and carried the cre-recombinase but not the *Oxr1* floxed allele (WT/Cre+/dose) did not show any reduction in Oxr1 expression (Figure 4B). The inducible knockout (Hom/Cre+/dose) mice were monitored closely, and it became apparent that, from approximately 25 days after tamoxifen dosing, animals began to display an ataxic gait (Supplementary Video S1). None of the control animals, such as those of the same genotype but not dosed with tamoxifen (Hom/Cre+/no dose), or dosed animals carrying only one copy of the conditional allele (Het/Cre+/dose), showed any gait disturbance or adverse effects.

To next quantify this phenotype, which likely influences motor function, behavioural analysis on the accelerating rotarod was carried out. As a measure of motor coordination, these data showed consistent performance from all control groups prior to dosing at 2 and 4 weeks post-tamoxifen treatment (Figure 4E). Interestingly, the inducible knockout group (Hom/Cre+/dose) displayed a significant drop in performance after 4 weeks, although it did not show any sign of motor dysfunction at 2 weeks after dosing (Figure 4E). Next, to determine whether the ataxia and reduced motor co-ordination were caused potentially by cell death in the brain, TUNEL staining was carried out on animals 32 days after tamoxifen administration; this was designated as disease end-point in Hom/Cre+/dose animals. Surprisingly, the pathology observed in Hom/Cre+/dose mice was highly reminiscent of *Oxr1* KO mice at P22, with apoptotic cells in the GCL of the cerebellum (Figure 4F). Together, we demonstrate for the first time there is intrinsic vulnerability in the cerebellum when Oxr1 is almost completely knocked down or deleted, irrespective of developmental age.

### 3.4. Pathogenic Variants in OXR1 That Cause Contrasting Clinical Outcomes Retain Neuroprotective Properties

**Figure 5:**
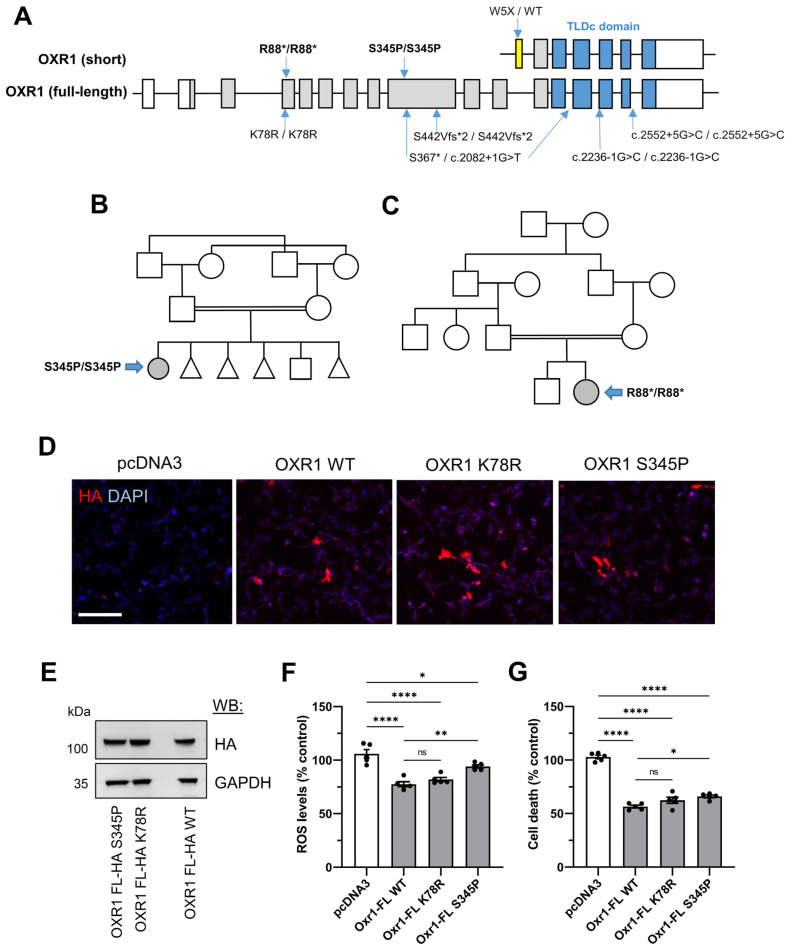
Analysis of human homozygous *OXR1* missense variants. (**A**) Diagram of published *OXR1* variants in full length (NM_001198533.2) and short (NM_001198534.1) isoforms, not to scale. Blue indicates position of the TLDc domain, and yellow indicates the unique first coding exon of the short isoform. Variants are K78R/K78R [35], S367*/c.2082+1G>T, S442Vfs*2/S442Vfs*2 and c.2236-1G>C/c.2236-1G>C [7], W5X [36] and c.2552+5G>C/c.2552+5G>C [8], with those described in this paper, S345P/S345P and R88*/R88*. (**B**,**C**) Family pedigrees of two novel OXR1 variants with probands indicated (grey filled symbol). (**D**) Representative images of SH-SY5Y cells transfected with the constructs indicated and immunostained with anti-HA. Scale bar: 200 µm. (**E**) Western blotting (WB) demonstrating the expression of OXR1 from SH-SY5Y cells transfected with the constructs and antibodies indicated. (**F**) SH-SY5Y cells transfected with the constructs shown treated with 1200 μM H_2_O_2_ for 24 h. (**G**) SH-SY5Y cells transfected with the constructs shown treated with 600 μM H_2_O_2_ for 24 h. Data are shown as +/− SEM, one-way ANOVA, *p*-values: * *p* ≤ 0.05, ** *p* ≤ 0.01, **** *p* ≤ 0.0001.

In addition to pathology in common with the *Oxr1* KO mouse model, the homozygous total loss-of-function variants in human *OXR1* described to date share severe, neurodevelopmental and neuropathological features [7,8]. Here, we present the description of two new variants that add to the clinical spectrum (Figure 5A). The first individual is a girl born to consanguineously married parents of Pakistani origin (Figure 5B) with an uneventful birth. The mother had a previous history of three early miscarriages due to IUGR. Developmentally, motor milestones were normally achieved (sitting at six months, walking at one year), but there was a significant speech lag with babbling around eighteen months, one-word speech around three years and three-word sentences at four years of age. At aged nine, there was no speech ability, and no visual contact was possible from the age of six. Seizure onset was recorded at the age of four-and-a-half; initially, myoclonic jerks were associated with walking difficulty and frequent falls. Multiple and various types of fits were described (generalized, tonic–clonic and focal), and seizures have not been controlled with sodium valproate, Carbamazepine and Levetiracetam. One hospital admission occurred at the age of eight due to pneumonia and increased frequency of seizures. Along with seizures, motor milestone regression has been progressive from the age of five years, and she is currently bed-bound (GMFCS-IV). Brain MRI carried out at 4 years of age appeared normal. Exome sequencing identified a homozygous missense variant in *OXR1* NM_001198533.2:c.1033T>C (p.Ser345Pro) that was confirmed by Sanger sequencing to have been inherited from both parents (Supplementary Figure S2). The second homozygous variant is a stop-gain at amino acid position 88 NM_001198533.2:c.262C>T (p.Arg88Ter) identified by whole genome sequencing in a female born to consanguineous parents (Figure 5C). At the time of medical examination, aged 1 year, the individual displayed global developmental delay, hypotonia and dystonia. Brain MRI and metabolic tests at the time were reportedly unremarkable.

Biological samples are not available for protein studies from either of the individuals; thus, there is no evidence that a truncated OXR1 protein is expressed with an N-terminal deletion as a consequence of the Arg88Ter variant. However, we wanted to investigate the potential influence of the Ser345Pro missense mutation by assessing OXR1 expression as well as quantifying the antioxidant activity of the mutant protein in the context of an oxidative stress-induced cell death assay that has been used previously to compare mutants in neuronal cells [2]. We compared this variant to the only other homozygous *OXR1* missense mutation reported that causes sensorineural hearing loss (c.233A>G, p.Lys78Arg) with no additional neurological involvement [35]. Both the S345P and K78R variants were detectable by immunocytochemistry after transfection in neuronal SH-SY5Y cells (Figure 5D). In addition, the expression of both mutants, when analysed by Western blot, was at a comparable level to WT OXR1, suggesting no overt effect on protein stability occurs due to the amino acid change (Figure 5E). Transfected SH-SY5Y cells were also subjected to hydrogen peroxide treatment (H_2_O_2_) to induce oxidative stress-associated cell death and reactive oxygen species (ROS) to assess the neuroprotective properties of OXR1. Compared to an empty vector control, the exogenous over-expression of WT human OXR1 significantly reduced cell death by approximately 50% and ROS levels by 25% (Figure 5F). Both disease-associated OXR1 mutants also conferred significant protection against oxidative stress, with reductions in cell death and ROS levels, although the S345P variant did not perform as well as the WT protein (Figure 5F). These findings suggest that these two variants are not acting as total loss-of-function alleles with respect to neuroprotection and antioxidant activity in this experimental context.

## 4. Discussion

Combining data from new genetic mouse models, this study has provided further insight into the importance of OXR1 for normal brain function. We reveal for the first time that cerebellar degeneration caused by ubiquitous loss of Oxr1 in vivo is independent of the developmental stage and that there is likely a critical threshold level of protein reduction that triggers neuroinflammation and eventual apoptosis. How exactly the lack of Oxr1 causes such a strikingly rapid inflammatory response and cell death is still unclear, although our data from neuronal-specific rescue support a cell-autonomous mechanism in neurons that drives the process.

Interestingly, the genes deregulated in the pre-symptomatic *Oxr1* KO cerebellum and their relative distribution show considerable overlap with cerebellar transcriptomics of lysosomal storage disorder (LSD) models, such as Niemann-Pick disease type-C [37]. Although the pattern of cerebellar pathology in *Npc1* loss-of-function mouse mutants is distinct from *Oxr1* KOs, with Purkinje cell death the predominant feature, RNA-seq studies have highlighted complement and glial cell activation alongside lysosomal enzymes as key deregulated genes [37,38]. Indeed, the most significant differentially expressed genes from our *Oxr1* KO study (Figure 1) overlap completely with those described as upregulated in *Npc1* mutants, such as *Ctsd*, *Lyz2*, *Tyrobp* and *C1q* [37,38]. These genes likely partially represent common markers of inflammation, yet their expression is also increased in other LSD mouse models [39]. One particularly relevant example comes from single-cell (sc)RNA-seq of both pre-symptomatic and symptomatic *Npc1*^−/−^ cerebellar tissue [40]. These data identified overlapping transcriptomic profiles in Purkinje cells and other cerebellar neuronal populations; for example, *Ctsd* and *Lyz2* were found to be significantly upregulated in Purkinje cells from *Npc1*^−/−^ symptomatic cerebellar tissue, but also in basket neurons and unipolar brush cells [40]. Interestingly, DEGs were only detected in microglial and endothelial populations at the asymptomatic time point, suggesting microglial activation precedes neuronal dysfunction. Furthermore, the non-neuronal cell transcriptomic profile largely overlapped with that of cerebellar neuronal cell types in symptomatic *Npc*^−/−^ mice, thus limiting the insights into the selective neuronal vulnerability of Purkinje neurons in this model [40]. Therefore, similar scRNA-seq analysis on Oxr1 KO could help better understand the relative contribution of neuronal and glial cell states to the pathology observed in Oxr1 KO cerebellum, in particular given the significant level of OXR1 expression in non-neuronal cells [32].

The Purkinje cell degeneration of the *Npc1*^−/−^ mutant is not shared with any of the various loss-of-function *Oxr1* KO lines. One suggestion is that the disease end-point is so rapid that ‘later onset’ cerebellar pathology, which might include additional regions of the cerebellum, cannot be analysed. Secondly, the related TLDc protein *Ncoa7* is highly expressed in Purkinje cells, whereas *Oxr1* is found mainly in the granule cell layer. Thus, we have proposed that *Ncoa7* is able to compensate for the loss of *Oxr1* in Purkinje cells [10]. Nonetheless, our bulk transcriptomic data described here highlight pathways that reflect the interaction of multiple cell types in intact brain tissue; as such, the differentially expressed genes are distinct from the recent studies of OXR1 patient-derived iPSCs and the subsequent three-dimensional neuronal cultures [8]. This likely stems from the differing origin and topographical organisation of the two model systems; the cerebral organoids derived from patient iPSCs demonstrated many fundamental, early neurodevelopmental differences compared to a control line [8], and it is not yet clear how these data relate to the specific postnatal neurodegeneration observed in humans and mice lacking OXR1.

Cell-autonomous neurotoxicity in the *Oxr1* KO mouse is suggested by pathological rescue of the phenotype over the long term using an Oxr1 cDNA transgene driven by a neuronal promoter. To provide more conclusive evidence for this hypothesis and to investigate the role of glial cells in the process, it would be necessary to carry out additional targeted rescue experiments in vivo. For example, multiple detailed and complementary studies support the occurrence of cell-autonomous neurodegeneration in *Npc*^−/−^ mice [41]; first from a similar prion promoter-driven cDNA replacement [42], followed by a chimeric mouse study, where the cerebellum contained a mixture of both wild-type and mutant *Npc*^−/−^ cells [43]. Here, wild-type neurons survived among a mixed *Npc*^+/+^ and *Npc*^−/−^ glial population, but *Npc*^−/−^ neurons did not survive. Whether the striking microglial activation we observe in the *Oxr1* KO mice reflects a protective or exacerbating response remains to be elucidated, but it would be valuable to extend the *Oxr1* conditional deletion studies to non-neuronal cell populations.

One common functional hypothesis that links TLDc proteins has begun to emerge, with the discovery of direct interaction with the vacuolar (v)-ATPase proton pump [11,44]. This multi-protein complex drives acidification of intracellular organelles and is composed of a cytosolic V_1_ domain responsible for ATP hydrolysis and a membrane V_0_ domain for hydrogen transport [45]. Association and dissociation of the V_1_ and V_0_ domains control v-ATPase activity, and a combination of molecular and structural studies have generated varied mechanistic hypotheses regarding the role of TLDc proteins in this essential process [46,47,48]. It is evident that the TLDc domain itself is the critical region for V-ATPase interaction and that amino acids conserved in all family members appear to be essential [11]. We have previously described a mouse *Ncoa7* knockout model that displays disrupted lysosomal functionality and lipid droplet formation in primary cells [10]. The hypothesis proposed was that the TLDc domain of NCOA7 facilitates the assembly or docking of the V1 subunits onto the V0 complex, potentially with the N-terminal LysM domain ‘tethering’ the protein to the membrane via association with phosphoinositide lipids [10]. Importantly, the full-length isoform of OXR1 also contains the same conserved LysM domain; thus, it is possible that v-ATPase functionality is similarly compromised when OXR1 is depleted. Interestingly, however, recent cryo-EM studies of the yeast OXR1 homologue (Oxr1p) point towards a disruptive rather than facilitatory role for the protein with respect to v-ATPase activity [46].

Data from loss-of-function *OXR1* patient fibroblasts suggest enhanced lysosomal biogenesis [7,49], and in models of lysosomal storage disease, there is evidence that this phenotype, coupled with upregulation of lysosomal enzymes, occurs as a compensatory mechanism for impaired lysosomal degradation and autophagy [37,40]. In addition, elevated cathepsin B has been described recently in OXR1 mutant fibroblasts [49]. Of note, we observe the upregulation of several lysosomal enzymes in the transcriptomic data from the *Oxr1* KO cerebellum, as well as the presence of aberrant lipid droplets and membranous structures by EM that are also indicative of dysfunction in and around the endo-lysosomal and autophagy systems. How these pathways are interrelated in the context of normal and disrupted OXR1 function requires further study, in particular given the striking overlap in clinical outcomes related to TLDc proteins and v-ATPase subunit variants in disease, including seizures, cerebellar pathology and hearing loss [50,51,52].

Our description of two new disease-causing *OXR1* variants adds to the small number published to date. Perhaps somewhat surprisingly, the homozygous S345P case shares more clinical features with the known loss-of-function alleles, as opposed to the hearing loss caused by the homozygous missense K78R mutation. We show here that OXR1 containing either one of these changes can be over-expressed and maintains the ability to protect against oxidative stress-induced cell death; as such, this specific antioxidant function may not be driving the clinical presentation. We were not able to determine protein expression data for the new OXR1 variants from patient cells, but one hypothesis might be that the S345P missense mutation causes endogenous protein instability; of note, this phenomenon has been reported for certain TBC1D24 variants outside of the TLDc domain [53,54]. However, it is assumed that expression of the short TLDc domain-containing OXR1 isoform (Figure 5A) will not be influenced by the missense and nonsense variants described here and may be able to partially functionally compensate for disruption of full-length OXR1 in certain contexts. Indeed, the importance of the short OXR1 isoform in neurodevelopment is supported by a familial heterozygous stop-gain variant associated with specific language impairment (NM_001198534: c.G15A:p.W5X) [36]. Furthermore, the deletion of exclusively a full-length, exclusively brain-expressed isoform in mice leads to a relatively subtle phenotype with no neurodegenerative features [24]. Thus, it is noteworthy that the human stop-gain R88X variant described here causes neurodevelopmental delay. We are not able to determine whether an N-terminal truncated OXR1 protein is initiated downstream from the R88 position, yet this particular variant supports the hypothesis that the full-length isoform of OXR1 is essential for normal development in humans. It will be intriguing to discover how all of the mammalian TLDc domain-containing proteins and their corresponding isoforms play common, unique and even competitive functional roles in normal brain function that might help delineate their exact roles in human disease.

## 5. Conclusions

In summary, our data demonstrate that the complete loss of all OXR1 isoforms influences lysosomal and neuroinflammatory pathways in the mammalian brain, with the inflammation likely driven by disruption of the gene in neurons. Furthermore, the associated cerebellar neurodegeneration and ataxia in the mouse *Oxr1* knockout occurs irrespective of developmental stage, revealing a fundamental role for the gene in the adult brain. Finally, we add to the spectrum of causative OXR1 human variants; further work is needed to establish the mechanistic link between these mutant alleles and the ataxia, seizures and neurodevelopmental phenotypes observed.

## Data Availability

Raw, unprocessed RNA-seq data files are available as NCBI accession numbers SAMN40425656 to SAMN40425667.

## Supplementary Materials

The following supporting information can be downloaded at: https://www.mdpi.com/article/10.3390/antiox13060685/s1, Supplementary Figure S1: Pathway analysis of differentially expressed genes in *Oxr1* KO cerebellar tissue. Reactome and KEGG pathway functional enrichment using a q-value ranked list to perform an ordered query using Gprofiler. Results are presented as negative log_10_ scale of adjusted enrichment *p*-values. Supplementary Figure S2: Sanger sequencing traces of OXR1 NM_001198533.2:c.1033T>C (p.Ser345Pro) variant. Supplementary Table S1: Ranked list of all differentially expressed genes comparing *Oxr1* WT and KO cerebellar tissue at P19 based on normalised RPKM values. Supplementary Video S1: Footage of Hom/Cre+/dose and Het/Cre+/dose littermate females 28 days after tamoxifen dosing.
