# Supplementary material for: Neuroinflammation and Lysosomal Abnormalities Characterise the Essential Role for Oxidation Resistance 1 in the Developing and Adult Cerebellum
Source: Antioxidants (Basel). 2024 Jun 3;13(6):685. doi: 10.3390/antiox13060685 (PMC11201099; doi:10.3390/antiox13060685)

Full unedited gel for Figure 3A

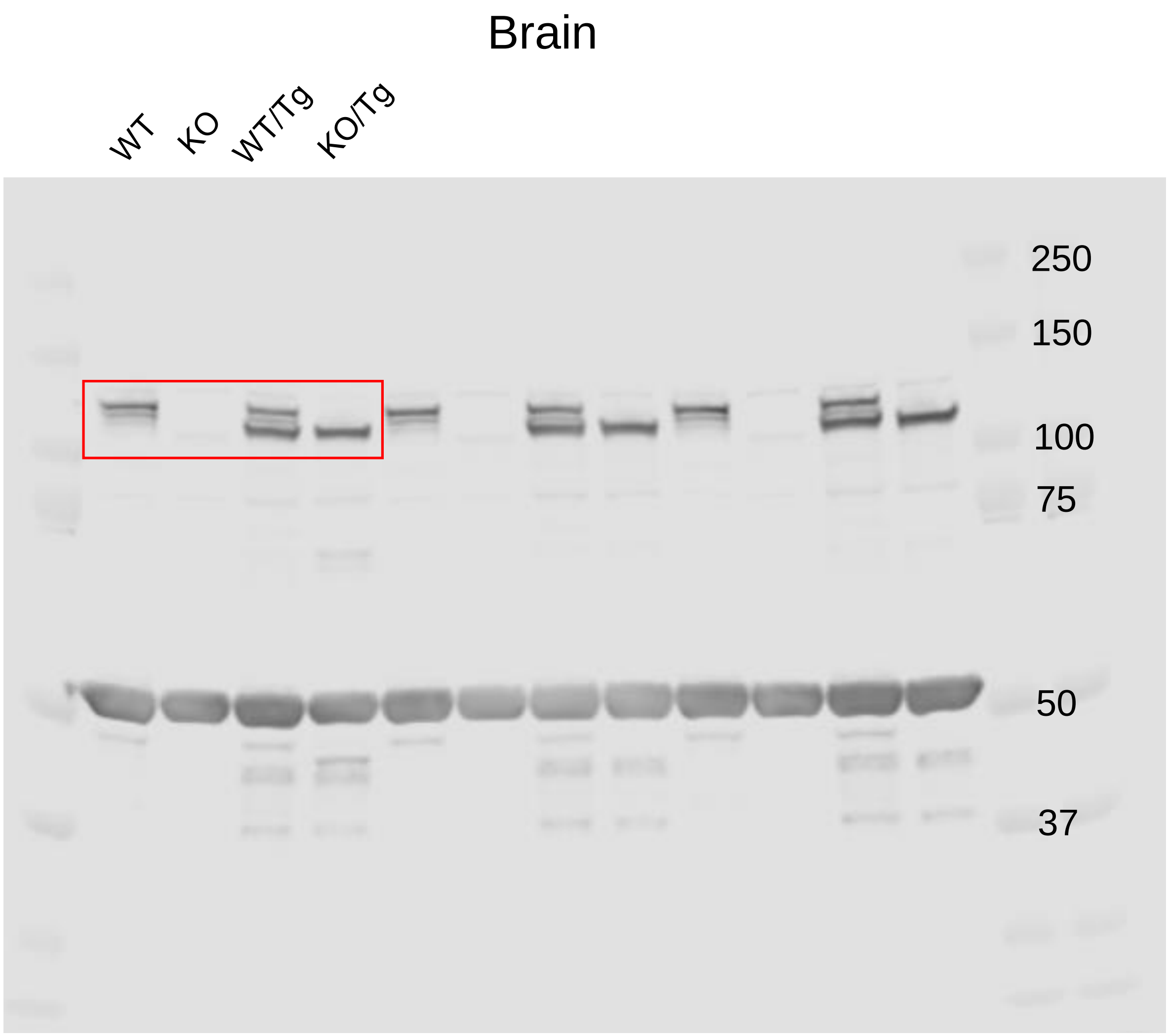

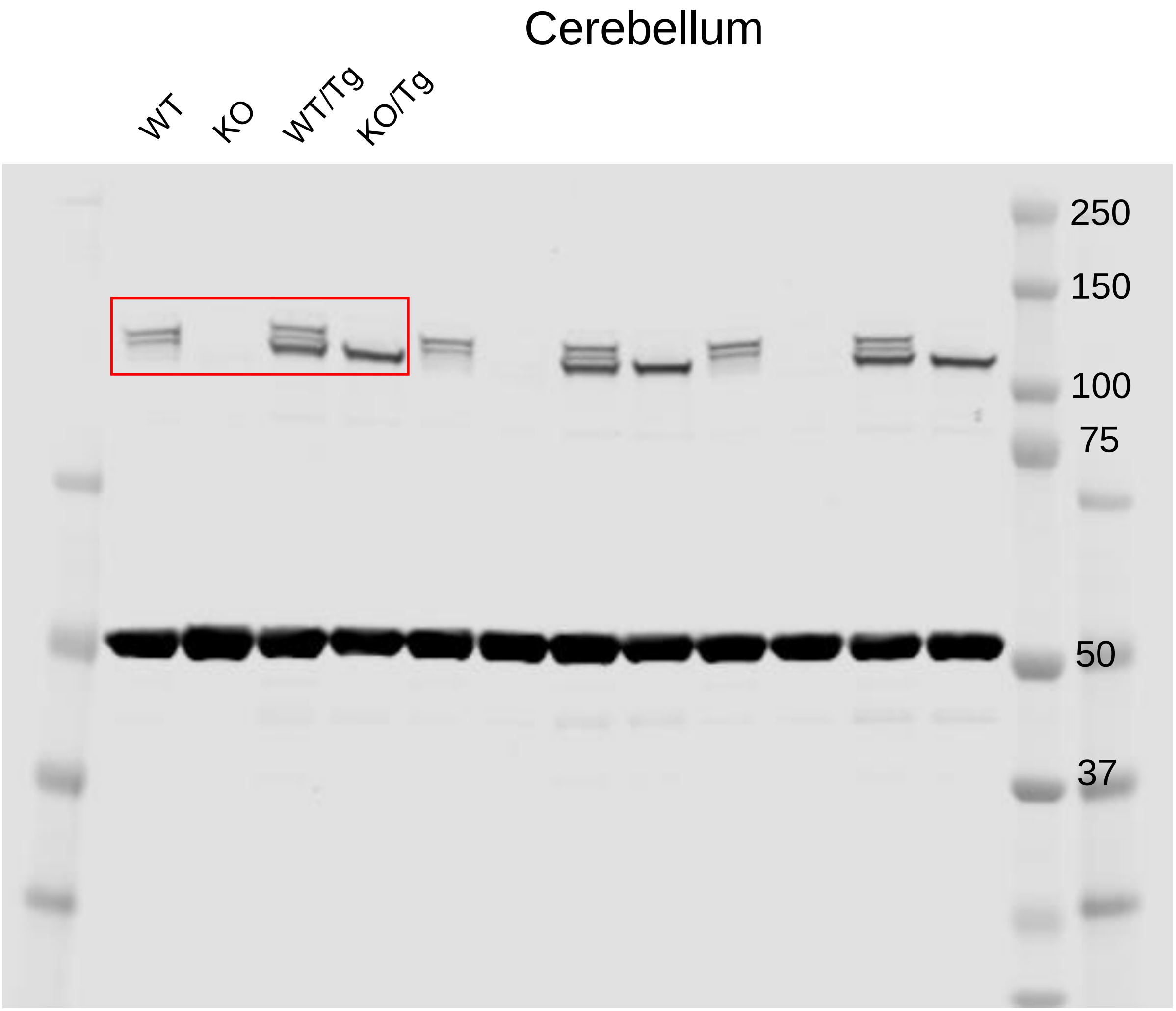

Full unedited gel for Figure 4B

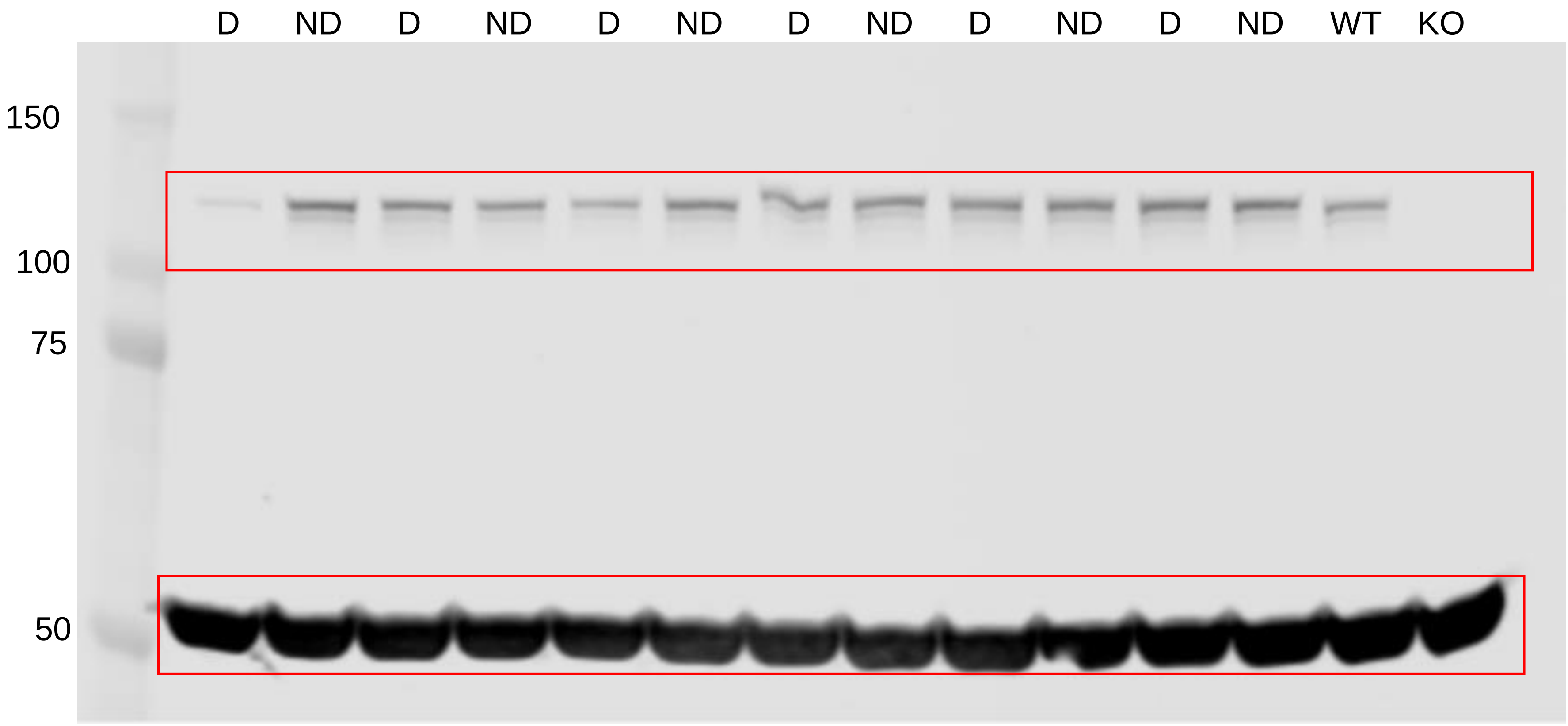

Full unedited gels for Figures 4C and 4D

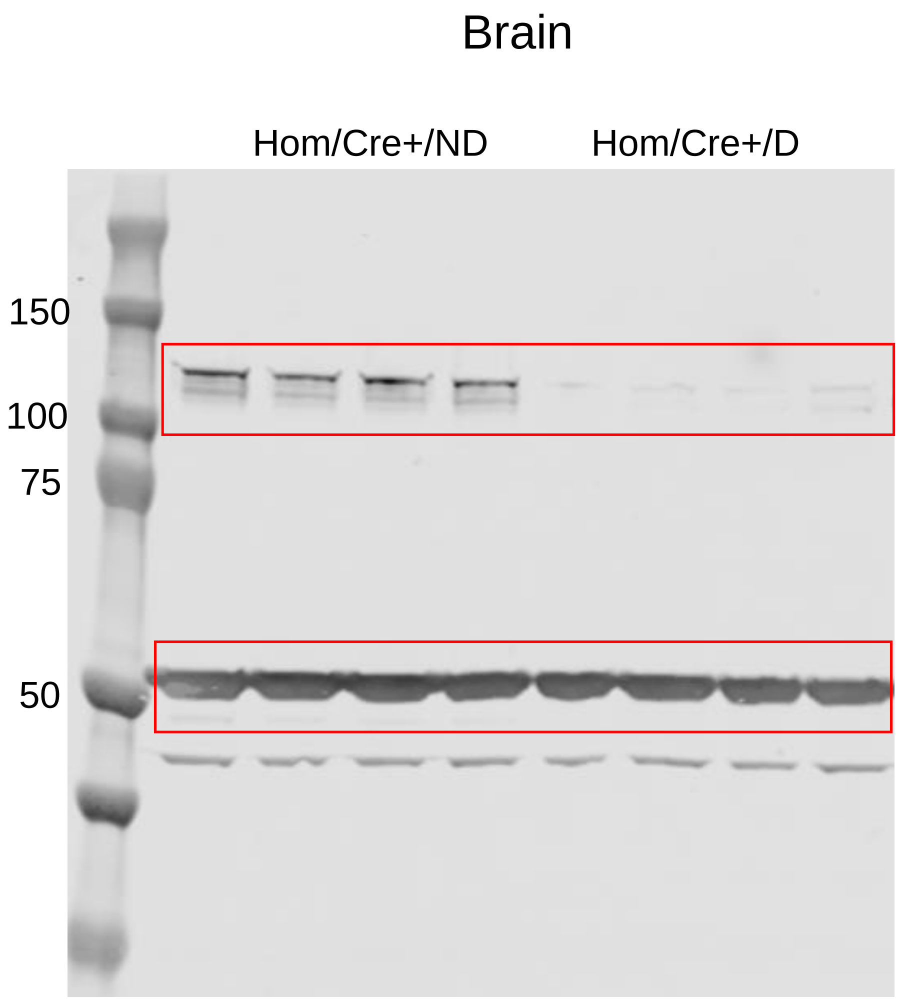

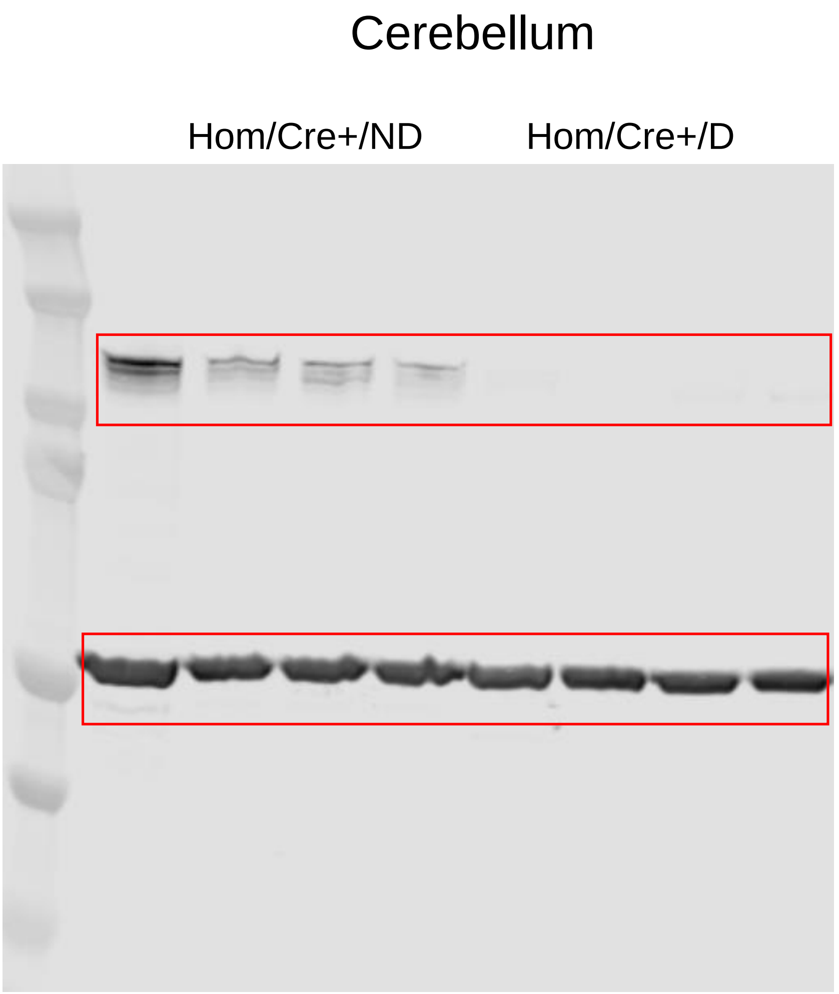

Full unedited gel for Figure 5E

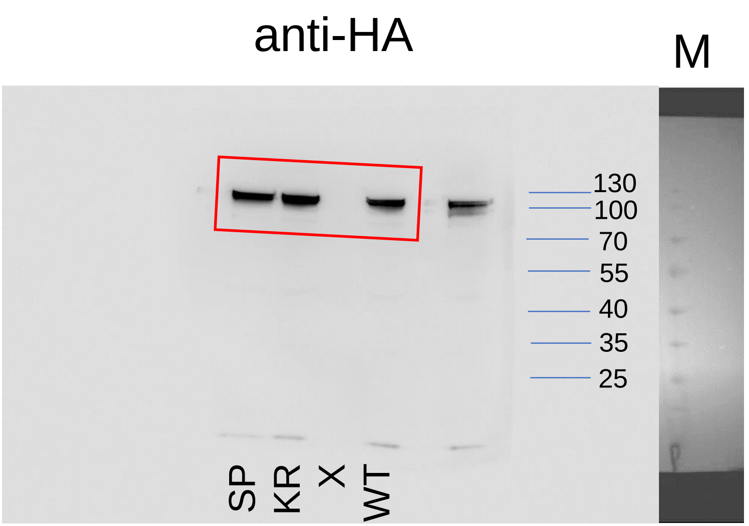

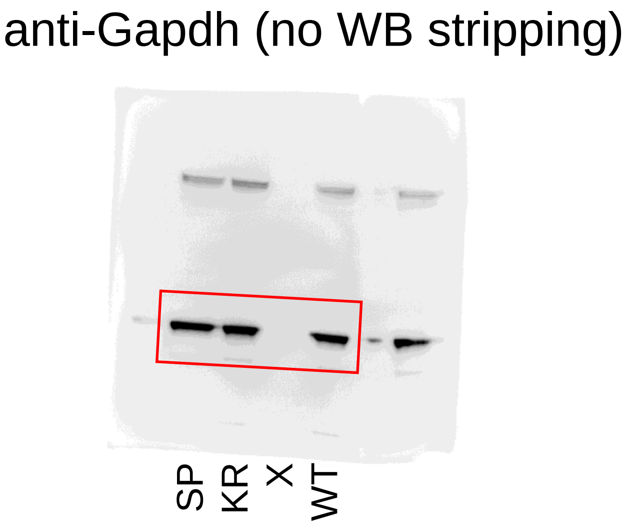

Supplement: Supplementary file 1 [file antioxidants-13-00685-s001.zip › Bucknor et al Full Westerns REV.pdf]
