# Supplementary figures and images for: Neuroinflammation and Lysosomal Abnormalities Characterise the Essential Role for Oxidation Resistance 1 in the Developing and Adult Cerebellum
Source: Antioxidants (Basel). 2024 Jun 3;13(6):685. doi: 10.3390/antiox13060685 (PMC11201099; doi:10.3390/antiox13060685)

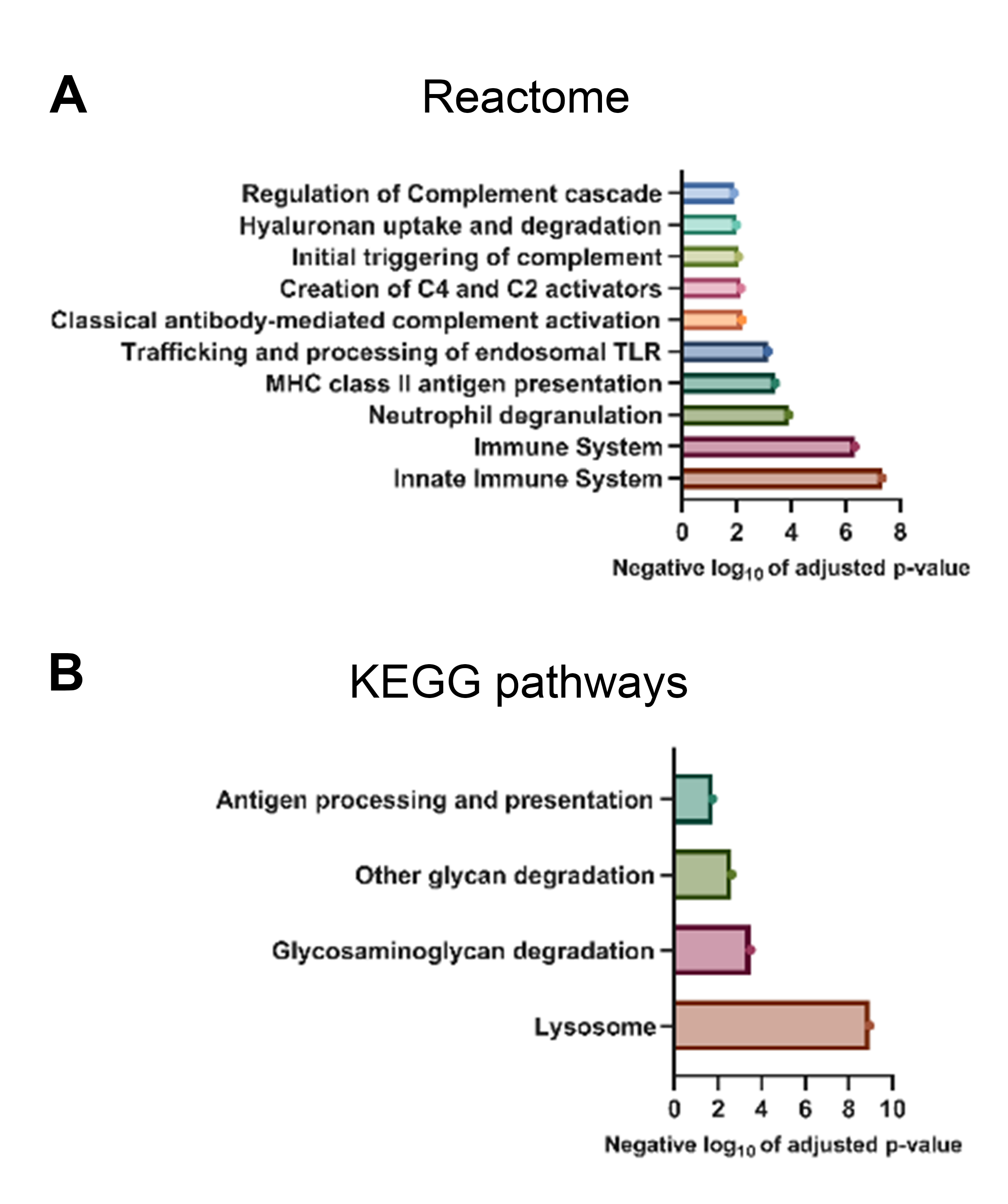

Supplement: Supplementary file 1 [file antioxidants-13-00685-s001.zip › Suppl Figure S1 flat 600dpi.tif]

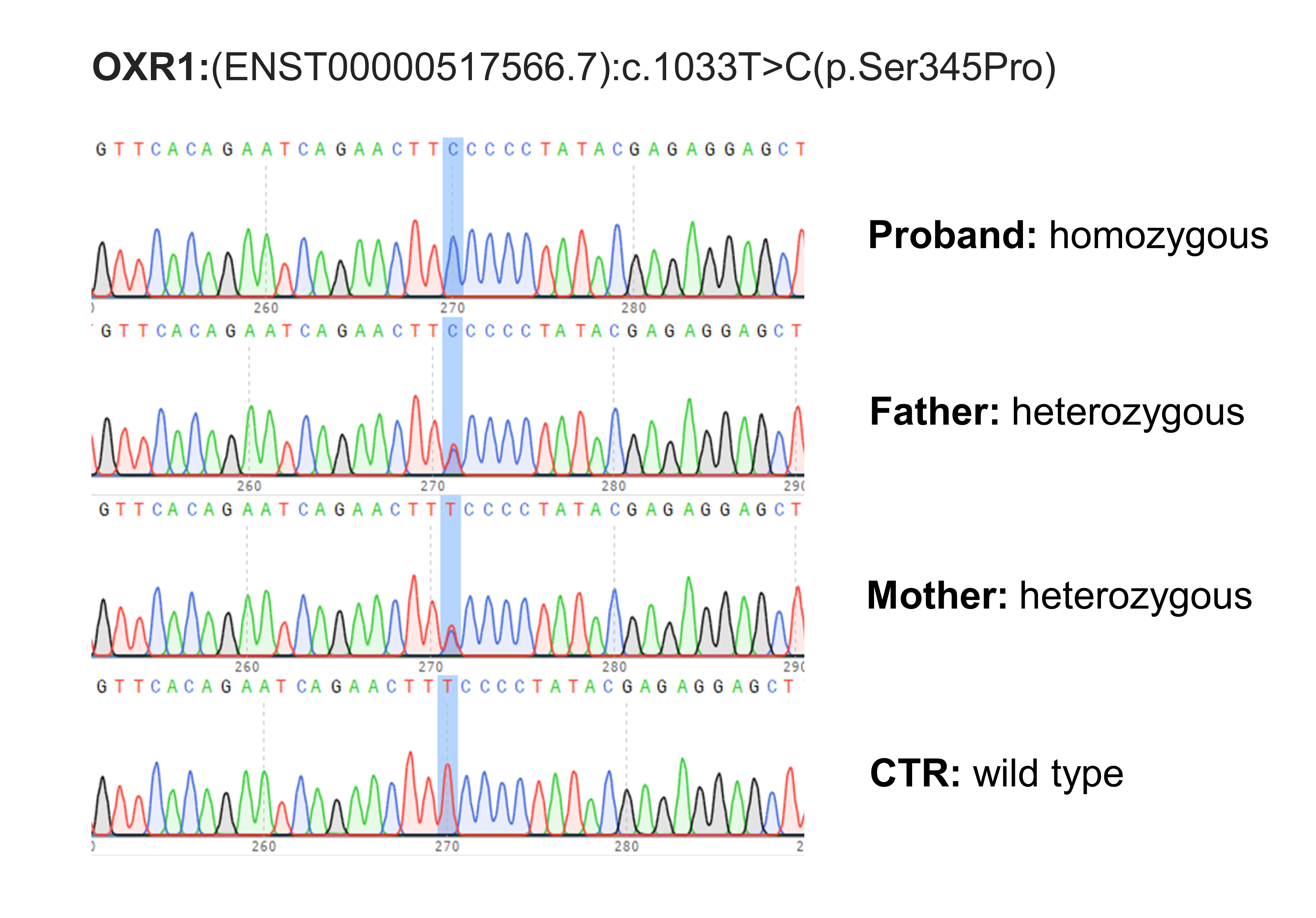

Supplement: Supplementary file 1 [file antioxidants-13-00685-s001.zip › Suppl Figure S2 flat 600dpi.tif]
